# Knockdown of TMEM160 leads to an increase in reactive oxygen species generation and the induction of the mitochondrial unfolded protein response

**DOI:** 10.1002/2211-5463.13496

**Published:** 2022-10-20

**Authors:** Kosei Yamashita, Misa Haraguchi, Masato Yano

**Affiliations:** ^1^ Department of Medical Technology, Faculty of Health Sciences Kumamoto Health Science University Japan

**Keywords:** mitochondria, mitochondrial unfolded protein response, oxidative stress, reactive oxygen species, TMEM160

## Abstract

Transmembrane protein 160 (TMEM160) was recently reported to be localized to the mitochondrial inner membrane, but mitochondrial function was noted to be unaffected by loss of *TMEM160*. In contrast to these previously published findings, we report here that the absence of TMEM160 influences intracellular responses. After confirming that TMEM160 is localized in the inner mitochondrial membrane, we knocked down TMEM160 in human cultured cells and analyzed the changes in cellular responses. TMEM160 depletion led to an upregulation of the mitochondrial chaperone HSPD1, suggesting that depletion induced the mitochondrial unfolded protein response (UPR^mt^). Indeed, the expression of key transcription factors that induce the UPR^mt^ (ATF4, ATF5, and DDIT3) was increased following TMEM160 depletion. Expression of the mitochondrial protein import‐receptors TOMM22 and TOMM20 was also enhanced. In addition, we observed a significant increase in reactive oxygen species (ROS) generation following TMEM160 depletion. Glutathione *S*‐transferases, which detoxify the products of oxidative stress, were also upregulated in TMEM160‐depleted cells. Immunoblot analysis was performed to detect proteins modified by 4‐hydroxynonenal (which is released after the peroxidation of lipids by ROS): the expression patterns of 4‐hydroxynonenal‐modified proteins were altered after TMEM160 depletion, suggesting that depletion enhanced degradation of these proteins. HSPD1, TOMM22, ATF4, ATF5, and DDIT3 remained upregulated after ROS was scavenged by *N*‐acetylcysteine, suggesting that once the UPR^mt^ is induced by TMEM160 depletion, it is not suppressed by the subsequent detoxification of ROS. These findings suggest that TMEM160 may suppress ROS generation and stabilize mitochondrial protein(s).

## Graphical abstract

TMEM160 depletion led to an upregulation of the mitochondrial unfolded protein response (UPR^mt^) and the mitochondrial protein import‐receptors TOMM22 and TOMM20 (TOMs). Reactive oxygen species (ROS) generation and the expression of glutathione *S*‐transferases (GSTs) were also upregulated in TMEM160‐depleted cells. TMEM160 may be involved in suppressing ROS generation and preventing the degradation of some types of mitochondrial protein(s).

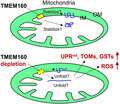

**Abbreviations**
4‐HNE: 4‐hydroxynonenal
ATF4 and ATF5: activating transcription factor 4 and 5, respectively
ATP5A: mitochondrial ATP synthase subunit alpha
CAT: catalase
DDIT3: DNA damage‐inducible transcript 3 (CHOP)
ETC: electron transport chain
GAPDH: glyceraldehyde‐3‐phosphate dehydrogenase
GSTA: glutathione *S*‐transferase alpha
HBSS: Hank's balanced salt solution
HSPA9: mitochondrial Hsp70
HSPD1: heat shock protein family D (Hsp60) member 1
LONP1: Lon peptidase 1
MTCO1: mitochondrially encoded cytochrome *c* oxidase I
NAC: *N*‐acetylcysteine
PRDX3: peroxiredoxin 3
ROS: reactive oxygen species
siRNA: small interfering RNA
SOD: superoxide dismutase
TMEM160: transmembrane protein 160
TOMM22: TOMM40, and TOMM20, translocase of outer mitochondrial membrane 22, 40, and 20, respectively
TXN2: thioredoxin 2
UPR^mt^
: mitochondrial unfolded protein response
UQCRC2: ubiquinol‐cytochrome *c* reductase core protein 2
VDAC1: voltage dependent anion channel 1

During the course of this study, another group reported that TMEM160 is a transmembrane protein localized to the mitochondrial inner membrane and that its loss does not affect mitochondrial function [1]. However, we demonstrated that the absence of TMEM160 influences intracellular responses.

Here, we verified that TMEM160 is a transmembrane protein of the mitochondrial inner membrane, supporting previously reported data [1]. Additionally, in contrast to the results reported the previous study, we found that various intracellular responses are induced by the lack of TMEM160. TMEM160 depletion led to upregulation of the mitochondrial unfolded protein response (UPR^mt^)‐related factor HSPD1 and increased levels of the transcription factors ATF4, ATF5, and DDIT3, which induce the UPR^mt^ [2]. Expression of the mitochondrial protein import receptors TOMM22 and TOMM20 was also enhanced by the lack of TMEM160. TMEM160 depletion led to increase in the levels of reactive oxygen species (ROS) and glutathione *S*‐transferases (GSTs), which detoxify the products of oxidative stress. Increased ROS generation enhances the peroxidation of lipids, and the resulting peroxidated lipids release 4‐hydroxynonenal (4‐HNE), which modifies proteins and DNA [3]; thus, we performed immunoblot analysis using anti‐4‐HNE antibody to detect 4‐HNE‐modified proteins. The expression of 4‐HNE‐modified proteins was altered after TMEM160 depletion, which suggested that these proteins had been degraded. Taken together, TMEM160 may be involved in suppressing ROS generation and preventing the degradation of some types of mitochondrial protein(s). Furthermore, when ROS was scavenged by *N*‐acetylcysteine (NAC), HSPD1, TOMM22, ATF4, ATF5, and DDIT3 remained upregulated, suggesting that depletion of TMEM160 induces the UPR^mt^ independently of subsequent detoxification of ROS by NAC.

## Materials and methods

### Reagents and antibodies

All reagents were purchased from Sigma‐Aldrich (St. Louis, MO, USA), Wako (Osaka, Japan), or Takara (Kyoto, Japan), unless otherwise stated. Anti‐DYKDDDDK antibody (Wako), anti‐TMEM160 antibody (Invitrogen, Carlsbad, CA, USA), anti‐VDAC1 antibody (Calbiochem‐Novabiochem, San Diego, CA, USA), anti‐HSPA9 (Sigma‐Aldrich), anti‐TOMM22 (Tom22) antibody [4], anti‐HSPD1 antibody (Sigma‐Aldrich), anti‐glyceraldehyde‐3‐phosphate dehydrogenase (anti‐GAPDH) antibody (Novus Biologicals, Littleton, CO, USA), anti‐LONP1 antibody (Sigma‐Aldrich), and Total OXPHOS Rodent WB Antibody Cocktail (Abcam, Cambridge, UK) containing anti‐ATP5A, UQCRC2, and MTCO1 antibodies were used to immunostain the cells and/or in immunoblot analysis. The anti‐TOMM40 (Tom40) and anti‐TOMM20 antibodies were obtained previously [5, 6]. Anti‐ATF4, ATF5, and DDIT3 antibodies were purchased from Proteintech (Rosemont, IL, USA).

### HeLa cell culture and isolation of stable transformants

HeLa cells were cultured in a growth medium (Dulbecco's modified Eagle's medium containing 10% fetal calf serum) at 37 °C in a 5% CO_2_ atmosphere. pCMV6‐TMEM160‐Myc‐DYKDDDDK (OriGene, Rockville, MD, USA), which expresses the TMEM160‐Myc‐DYKDDDDK protein with a DYKDDDDK‐tag at the C‐terminus, was transfected into HeLa cells using Lipofectamine 3000 (Invitrogen) and Opti‐MEM I (Gibco, Grand Island, NY, USA). Control cells were obtained by transfecting HeLa cells with pCMV6‐Entry (OriGene), an empty vector. After 72 h of culture, the cells were harvested and suspended in a growth medium containing 1 mg·mL^−1^ G418. The cell suspension was diluted stepwise in a 96‐well plate. Each single colony resistant to G418 was selected and further cultured as a stable transformant. The cells were harvested in ice‐cold phosphate‐buffered saline (PBS), containing 1 mm ethylenediaminetetraacetic acid, and washed twice with PBS. The cells were lysed with PBS containing 1% Triton X‐100 and centrifuged at 10 000 × ***g*** for 5 min. The supernatant was mixed with sodium dodecyl sulfate‐polyacrylamide gel electrophoresis (SDS/PAGE) loading buffer for immunoblotting analysis to evaluate the expression of TMEM160‐Myc‐DYKDDDDK.

### Immunostaining of HeLa stable transformants

Using stable HeLa transformants cultured on coverslips, triple staining with Alexa Fluor488, Mito Tracker Red, and Hoechst dye was performed to detect TMEM160‐Myc‐DYKDDDDK. The cells were treated with a culture medium containing Mito Tracker Red (Invitrogen) and washed twice with Hank's balanced salt solution (HBSS). The cells were fixed with 4% paraformaldehyde and treated with PBS containing 1% Triton X‐100. The cells were further treated with an anti‐DYKDDDDK antibody, followed by treatment with the secondary antibody goat anti‐mouse immunoglobulin G (IgG) conjugated with Alexa Fluor488 (Abcam). The cells were then stained with the Hoechst dye (Sigma‐Aldrich). The fluorescence of Alexa Fluor488 (green), MitoTracker Red (red), and Hoechst dye (blue) was photographed using a fluorescence microscope.

### HepG2 cell culture, isolation of mitochondria, alkali‐ and digitonin‐extraction assay

HepG2 cells were cultured as described above, harvested in ice‐cold PBS containing 1 mm ethylenediaminetetraacetic acid, and washed twice with PBS. The cells were suspended in an isolation buffer [3 mm HEPES‐KOH (pH 7.4), 0.21 m mannitol, 0.07 m sucrose, 0.2 mm EGTA] and homogenized with a Dounce homogenizer (Wheaton, IL, USA) on ice. The homogenate was centrifuged at 500 × ***g***, and the supernatant containing mitochondria was collected. The supernatant was further centrifuged at 10 000 × ***g*** to collect the mitochondrial fraction as a pellet.

For the alkali extraction assay, the isolated mitochondria were sonicated and extracted with 0.1 m Na_2_CO_3_ (pH 11.5) as described previously [4]. After centrifugation at 100 000 × ***g***, the supernatant was collected as the mitochondrial soluble fraction, and the pellet was collected as the mitochondrial membrane fraction. To separate the fraction containing the mitochondrial outer membrane as the supernatant, the mitochondria were treated with an isolation buffer containing 0.1, 0.2, 0.4, or 0.8 mg·mL^−1^ digitonin, followed by centrifugation at 10 000 × ***g***. Each supernatant and pellet was mixed with the SDS/PAGE loading buffer for immunoblot analysis.

### Transfection of siRNA into HepG2 cells

Lipofectamine RNAiMAX (Invitrogen) and Opti‐MEM I (Gibco) were used to transfect small interfering RNAs (siRNAs) into HepG2 cells. The siRNAs used to knockdown TMEM160 mRNA (sense: 5′‐GUCAUCUCCUUCAUGCAGAdTdT‐3′, antisense: 5′‐UCUGCAUGAAGGAGAUGACdCdC‐3′) and universal negative control siRNA (sense: 5′‐UUCUCCGAACGUGUCACGUdTdT‐3′, antisense: 5′‐ACGUGACACGUUCGGAGAAdTdT‐3′) were purchased from JBioS (Saitama, Japan). After transfecting the siRNAs (final concentration of 48 nm), the cells were cultured for 72 h. To examine the effect of NAC‐treatment on the cells, NAC (final concentration of 10 mm, pH 7.5, adjusted by NaOH) was added to the culture medium after 24 h of siRNA‐transfection, and the cells were cultured for another 48 h. For immunoblot analysis, the HepG2 cells were harvested, washed, and lysed with PBS containing 1% Triton X‐100. After centrifugation at 10 000 × ***g***, the supernatant was mixed with the SDS/PAGE loading buffer.

### Immunoblot analysis

After SDS/PAGE, the proteins were transferred onto polyvinylidene difluoride membranes. The membranes were blocked with 5% skimmed milk and subjected to immunoblot analysis using enhanced chemiluminescence western blotting detection reagents (GE Healthcare, Buckinghamshire, UK), as described previously [4]. Immunoblotting was performed using the antibodies described above. Chemiluminescence images of the membranes were obtained using Ez‐Capture MG (ATTO, Tokyo, Japan). The intensity of the bands (protein expression levels) was analyzed using the imagej software (https://imagej.nih.gov/ij/).

### Quantitative polymerase chain reaction

Total RNA was isolated from HepG2 cells using TRIzol reagent (Invitrogen). The isolated RNA was reverse‐transcribed to cDNA using a PrimeScript RT reagent kit (Takara). Quantitative polymerase chain reaction (PCR) was performed using cDNA as the template on a LightCycler Nano system (Roche, Basel, Switzerland). The oligonucleotides used for quantitative PCR are listed in Table S1. The mRNA levels of the target gene were normalized to those of *GAPDH* mRNA.

### Measurement of ROS generation and apoptosis

HepG2 cells were cultured in microwell plates as described above. After siRNA transfection, the cells were cultured for 72 h and used to quantify ROS generation and detect apoptosis. To quantify ROS generation, the cells were treated with CM‐H_2_DCFDA (Invitrogen) and washed thrice with HBSS. The intensity of green fluorescence (excitation, 485 nm; emission, 535 nm) was measured using an Infiniti F200 Pro (TECAN, Zürich, Switzerland). To detect apoptotic cells, HepG2 cells were treated with JC‐1 (Dojindo, Kumamoto, Japan) and washed thrice with HBSS. The intensities of green fluorescence (excitation, 485 nm; emission, 535 nm) and red fluorescence (excitation, 535 nm; emission, 590 nm) were measured using Infiniti F200 Pro.

### Statistical analysis

Data were analyzed using Student's *t*‐test and reported as mean ± standard deviation (SD).

## Results

### Confirmation of mitochondrial localization of TMEM160

**Fig. 1:**
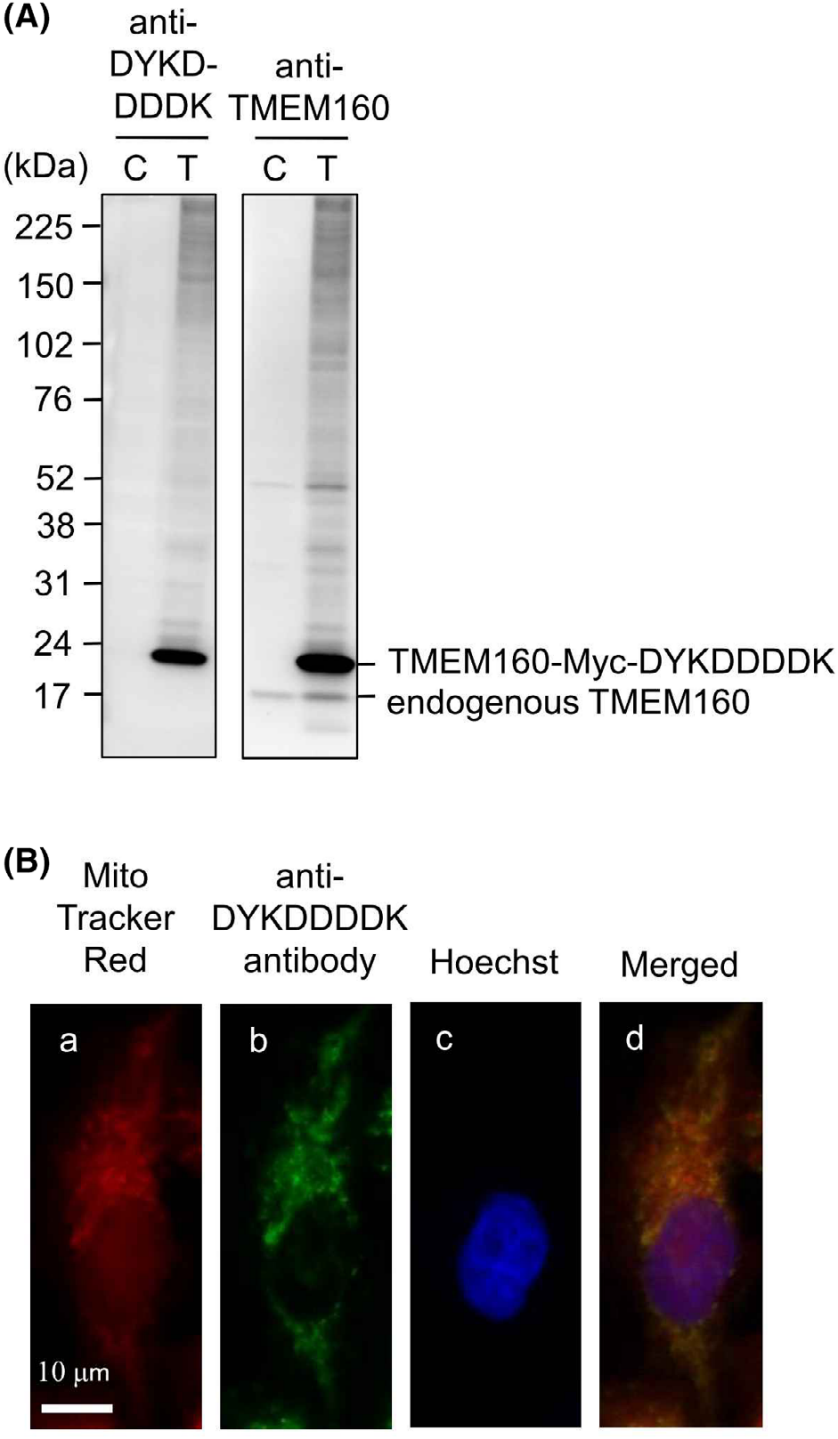
Confirmation of mitochondrial localization of TMEM160. (A) Stable transformants of HeLa cells transfected with control empty vector or vector expressing TMEM160‐MYC‐DYKDDDDK were lysed and subjected to immunoblot analysis [anti‐DYKDDDDK, anti‐DYKDDDDK antibody; anti‐TMEM160, anti‐TMEM160 antibody; C, control cell lysate; T, TMEM160‐MYC‐DYKDDDDK‐expressing cell lysate.]. (B) TMEM160‐Myc‐DYKDDDDK‐expressing HeLa cells were stained with Mito tracker red (a), anti‐DYKDDDDK antibody and secondary antibody conjugated with Alexa Fluor488 (b), and Hoechst (c); merged images of these three photograph are also shown (d). Scale bar, 10 μm.

A recent study reported that TMEM160 localizes to the mitochondrial inner membrane [1]. We evaluated the intracellular localization of endogenous TMEM160 in HepG2 and HeLa cells using immunostaining; however, we did not detect staining of endogenous TMEM160. Therefore, we attempted to construct a single line of HepG2 cells overexpressing TMEM160 by limiting the dilution method but failed to obtain these cells because HepG2 cells tend to adhere to each other. We next constructed a single cell line of HeLa overexpressing TMEM160. We successfully generated a single cell line of stably transformed HeLa cells containing a plasmid expressing TMEM160‐Myc‐DYKDDDDK, in which TMEM160 was C‐terminally fused with a Myc‐tag followed by a DYKDDDDK‐tag. HeLa cells stably transformed with pCMV6‐Entry (empty vector) were used as controls. To examine the expression of endogenous TMEM160 and TMEM160‐Myc‐DYKDDDDK, immunoblot analysis was performed using anti‐DYKDDDDK and anti‐TMEM160 antibodies (Fig. 1A). For the anti‐DYKDDDDK antibody, a strong band corresponding to TMEM160‐Myc‐DYKDDDDK was detected only in the lysate of TMEM160‐Myc‐DYKDDDDK‐expressing cells. For the anti‐TMEM160 antibody, a band corresponding to endogenous TMEM160 was detected in the lysates of both control and TMEM160‐Myc‐DYKDDDDK‐expressing cells. These results indicated that stable transformants of HeLa cells had been successfully generated. TMEM160‐Myc‐DYKDDDDK‐expressing HeLa cells were cultured on coverslips, treated with Mito Tracker Red, fixed with 4% paraformaldehyde, permeabilized with PBS containing 0.1% Triton X‐100, and subjected to immunostaining with anti‐DYKDDDDK antibody and anti‐mouse IgG conjugated with Alexa Fluor488, followed by staining with Hoechst dye (Fig. 1B). The fluorescence image of Alexa Fluor488 was almost identical to that of Mito Tracker Red, indicating that TMEM160‐Myc‐DYKDDDDK was localized in the mitochondria.

### Confirmation of mitochondrial inner membrane localization of TMEM160

**Fig. 2:**
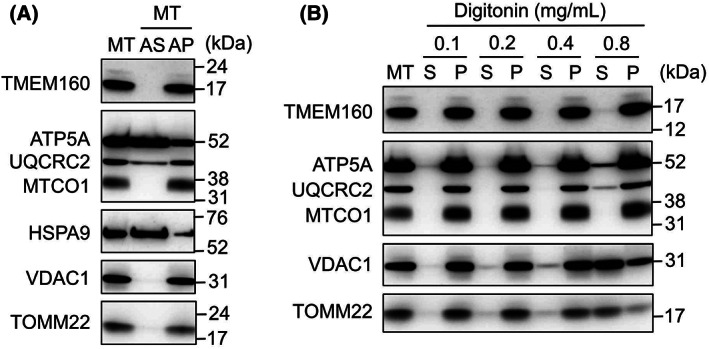
Confirmation of mitochondrial inner membrane localization of TMEM160. (A) Mitochondria were isolated from HepG2 cells as described in the “Materials and methods” and subjected to the alkali extraction assay. Immunoblot analysis was performed [AP, alkali‐resistant pellet fraction; AS, alkali‐soluble supernatant fraction; ATP5A, mitochondrial ATP synthase subunit alpha; HSPA9, mitochondrial Hsp70; MT, isolated mitochondria; MTCO1, mitochondrially encoded cytochrome c oxidase I; TOMM22, translocase of outer mitochondrial membrane 22; UQCRC2, ubiquinol‐cytochrome c reductase core protein 2; VDAC1, voltage‐dependent anion channel 1]. (B) the mitochondria isolated from HepG2 cells were treated with digitonin at the indicated concentration, followed by centrifugation to obtain the supernatant and pellet fractions [P, pellet fraction; S, supernatant fraction].

The mitochondria isolated from HepG2 cells were subjected to an alkali extraction assay using 0.1 m Na_2_CO_3_ (pH 11.5; Fig. 2A). The TMEM160 protein was detected in the alkali‐resistant pellet fraction along with MTCO1 [mitochondrially encoded cytochrome c oxidase I (transmembrane protein in inner membrane)], VDAC1 (transmembrane protein in outer membrane), and TOMM22 [translocase of outer mitochondrial membrane 22 (transmembrane protein in outer membrane)], whereas ATP5A [mitochondrial ATP synthase subunit alpha (inner membrane‐faced protein)] and HSPA9 [mitochondrial Hsp70 (soluble protein in matrix)] were mostly detected in the alkali‐soluble supernatant fraction. These results indicate that TMEM160 is a mitochondrial transmembrane protein that resides in either the outer or inner membrane. We next performed a digitonin extraction assay using the isolated mitochondria (Fig. 2B). When mitochondria were treated with 0.1 mg·mL^−1^ digitonin, all proteins were detected in the pellet, indicating that most of the mitochondria were intact. However, when mitochondria were treated with 0.8 mg·mL^−1^ digitonin, both VDAC1 and TOMM22, which reside in the mitochondrial outer membrane, were mainly detected in the supernatant, which suggested that these mitochondrial outer membrane proteins were partially solubilized by 0.8 mg·mL^−1^ digitonin. In contrast, TMEM160 was detected in the pellet, along with ATP5A and MTCO1, which reside on/in the mitochondrial inner membrane. The mitochondrial inner membrane is not solubilized by 0.8 mg·mL^−1^ digitonin. Thus, similarly to ATP5A and MTCO1, TMEM160 resides in the mitochondrial inner membrane. Taken together, these results confirm that TMEM160 is a mitochondrial transmembrane protein that resides in the inner membrane.

### Depletion of TMEM160 induced expression of the UPR^mt^ ‐related mitochondrial molecular chaperone HSPD1, mitochondrial protein import receptor TOMM22, and TOMM20

**Fig. 3:**
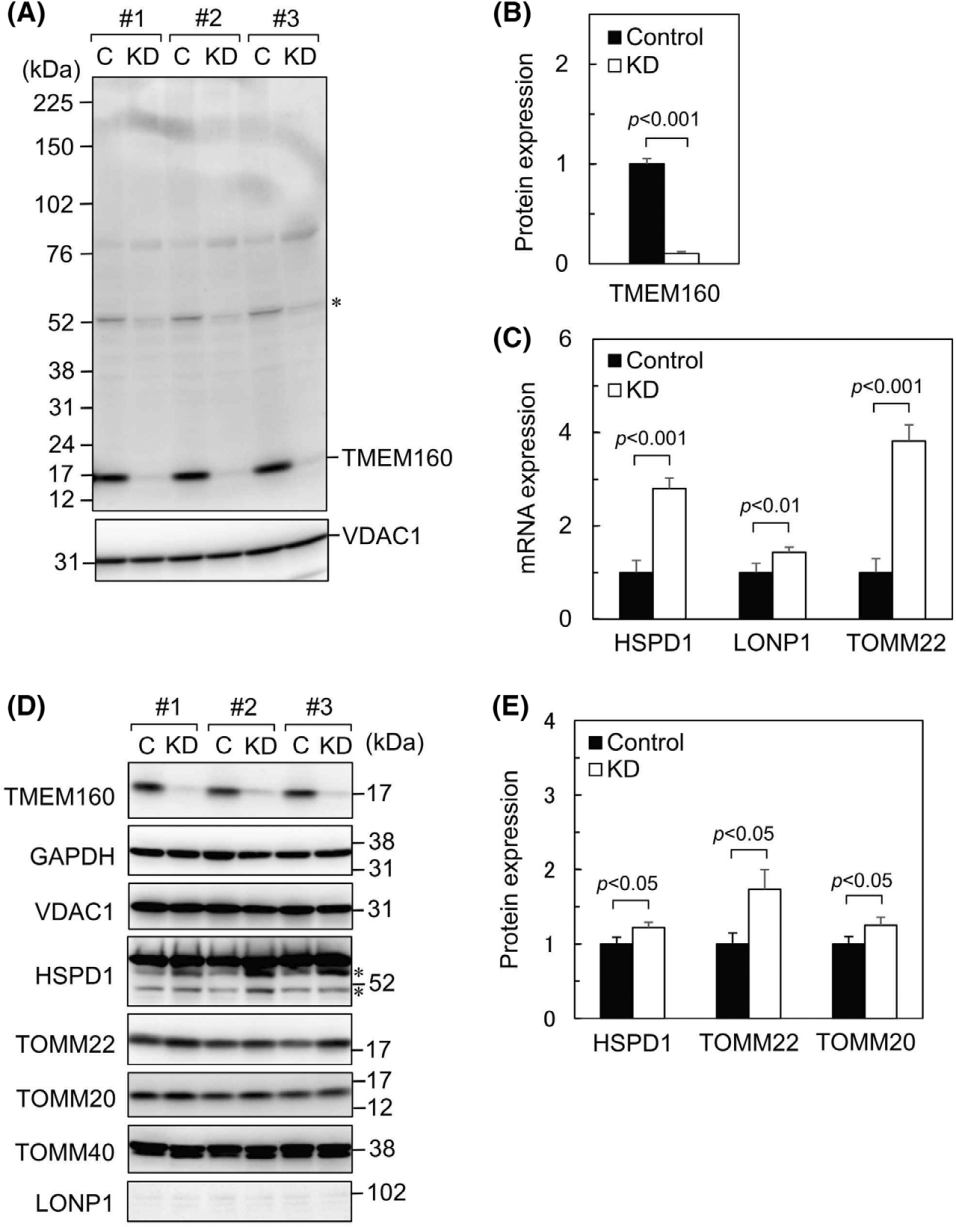
Depletion of TMEM160 led to induction of expression of the UPR^mt^‐related mitochondrial molecular chaperone HSPD1 and mitochondrial protein import receptors TOMM22 and TOMM20. HepG2 cells were transfected with control siRNA and TMEM160‐targeted siRNA. An asterisk indicates an unknown band that appeared at approximately 52 kDa; (A) proteins extracted from the cells were subjected to immunoblot analysis to determine TMEM160 protein levels (*n* = 3) [C, control cell lysate; KD, TMEM160‐depleted cell lysate]. (B) Protein expression of TMEM160 was quantified. data were analyzed using Student's *t*‐test, and the error bars represent mean ± standard deviation (SD). Protein expression levels of the control were set at 1. (C) mRNA levels of HSPD1, LONP1, and TOMM22 were examined using quantitative PCR (*n* = 4). Data were analyzed using Student's *t*‐test, and the error bars represent mean ± SD. mRNA levels of the control were set as 1 [HSPD1, heat shock protein family D (Hsp60) member 1; LONP1, Lon peptidase 1; TOMM22, translocase of outer mitochondrial membrane 22]. (D) the proteins extracted from cells were subjected to immunoblot analysis to determine expression levels of the indicated proteins (*n* = 3). Asterisks indicate bands corresponding to hypothetical degraded portions of HSPD1 [GAPDH, glyceraldehyde‐3‐phosphate dehydrogenase; TOMM40 and TOMM20, translocase of outer mitochondrial membrane 40 and 20, respectively]. (E) Protein expression of HSPD1, TOMM22, and TOMM20 was quantified. data were analyzed using Student's *t*‐test, and the error bars represent mean ± SD. Protein expression levels of the control were set at 1.

We examined the responses of HepG2 cells to TMEM160 knockdown. HepG2 cells were transfected with control siRNA and TMEM160‐targeted siRNA, and the protein levels of TMEM160 and VDAC1 were evaluated (Fig. 3A,B). A strong band (approximately 17 kDa), theoretically estimated to be endogenous TMEM160, was detected in the control cell lysate; this band was diminished upon using TMEM160‐targeted siRNA, which indicated that the band corresponded to endogenous TMEM160. However, unknown weak bands at approximately 52 kDa were also diminished by TMEM160‐targeted siRNA, suggesting that unknown protein(s) and/or TMEM160 itself bound to TMEM160 strongly enough to resist the effects of SDS/PAGE loading buffer containing sodium lauryl sulfate and 2‐mercaptoethanol. The protein levels of VDAC1 remained unchanged. These results suggest that the TMEM160‐targeted siRNA specifically knocked down TMEM160. Typically, when unfolded and/or misfolded proteins accumulate in mitochondria, the UPR^mt^ is activated, and the mitochondrial chaperone HSPD1 and protease LONP1 are imported into the mitochondria to refold and/or digest unfolded/misfolded proteins to maintain mitochondrial quality [7]. To examine whether TMEM160 depletion induced the UPR^mt^, we measured the mRNA levels of HSPD1 and LONP1 (Fig. 3C). The mRNA levels of the mitochondrial protein import receptor TOMM22 were also measured, as we previously found that TOMM22 was upregulated by the UPR^mt^ [8]. We found that the levels of these mRNAs were significantly elevated when TMEM160 was knocked down. Next, we examined their protein expression (Fig. 3D,E), including that of TOMM20, which is upregulated by the UPR^mt^ [2]. Results showed that HSPD1, TOMM22, and TOMM20 were upregulated, whereas LONP1 levels did not change after TMEM160 depletion. These findings indicate that the absence of TMEM160 weakly activates the UPR^mt^. To rule out off‐target effects, another siRNA for TMEM160 was used to deplete TMEM160 (Fig. S1). We observed a considerable reduction in the mRNA levels of TMEM160 and a substantial increase in those of HSPD1 and TOMM22. Morphological observation of mitochondria in both control and TMEM160‐depleted cells stained with MitoTracker Red and Hoechst revealed similar results between cells (Fig. S2).

### Depletion of TMEM160 upregulated the transcription factors ATF4, ATF5, and DDIT3, which induce UPR^mt^

**Fig. 4:**
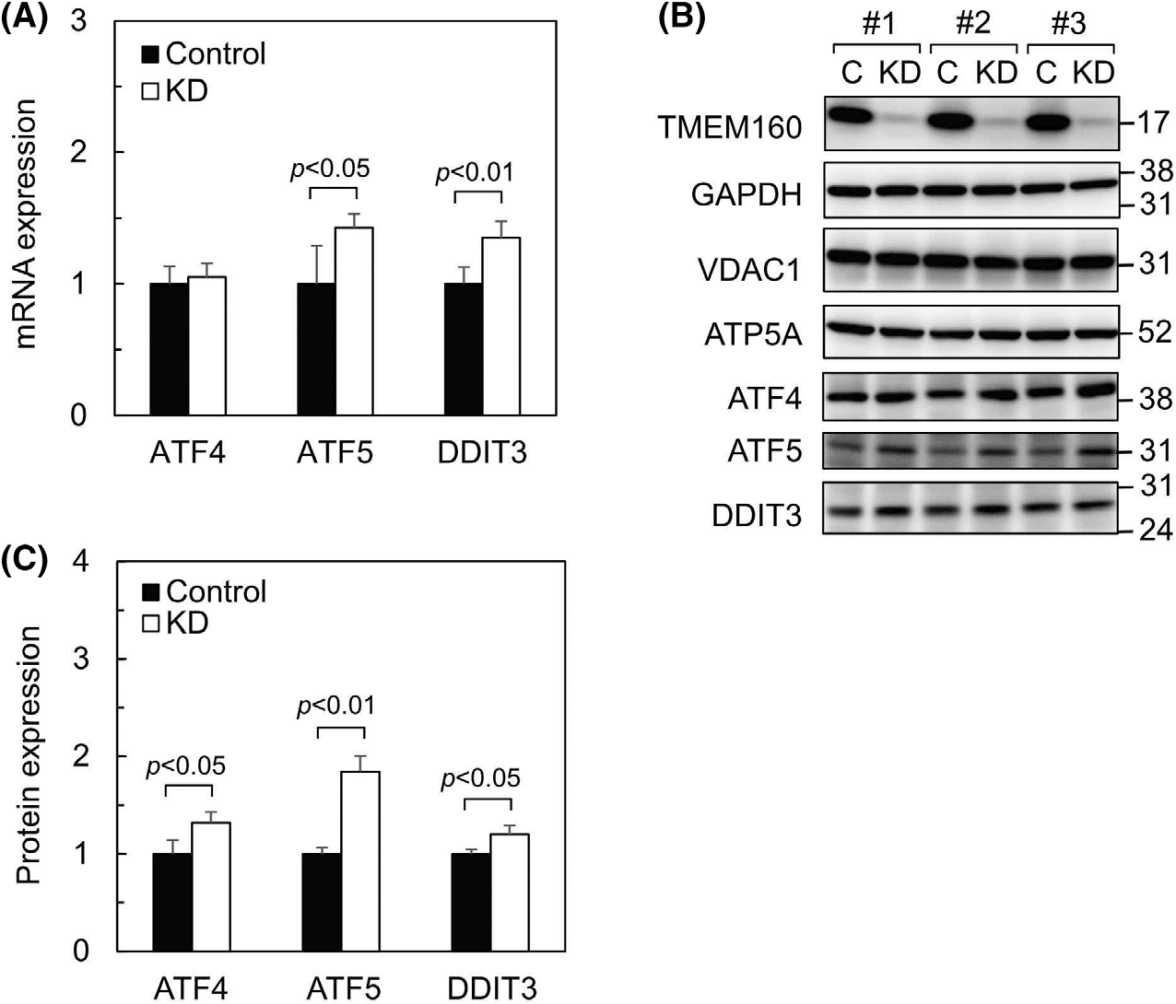
Depletion of TMEM160 upregulates the transcription factors ATF4, ATF5, and DDIT3, which induce the UPR^mt^. HepG2 cells were transfected with control siRNA and TMEM160‐targeted siRNA. (A) mRNA levels of ATF4, ATF5, and DDIT3 were measured using quantitative PCR (*n* = 4). Data were analyzed using Student's *t*‐test, and the error bars represent mean ± standard deviation (SD). mRNA levels of the controls were considered as 1 [ATF4 and ATF5, activating transcription factor 4 and 5; DDIT3, DNA damage‐inducible transcript 3 (CHOP)]. (B) Proteins extracted from the cells were subjected to immunoblot analysis to determine the expression levels of the indicated proteins (*n* = 3). (C) Protein expression of ATF4, ATF5, and DDIT3 was quantified. data were analyzed using Student's *t*‐test, and the error bars represent mean ± SD. Protein expression levels of the control were set at 1.

Damage to mitochondria by the accumulation of aggregated proteins enhances the phosphorylation of eIF2 alpha, followed by selective translation of UPR^mt^‐inducing transcription factors such as ATF4, ATF5, and DDIT3 [2]. To confirm whether the pathway that induces the UPR^mt^ is activated by a lack of TMEM160, we examined the expression levels of ATF4, ATF5, and DDIT3 (Fig. 4). We found that the mRNA (Fig. 4A) and protein levels (Fig. 4B,C) of some transcription factors were increased after TMEM160 depletion. These results suggest that TMEM160 depletion induces the UPR^mt^ depending on ATF4, ATF5, and/or DDIT3.

### Depletion of TMEM160 led to increase in ROS generation, GST gene expression, and protein degradation

**Fig. 5:**
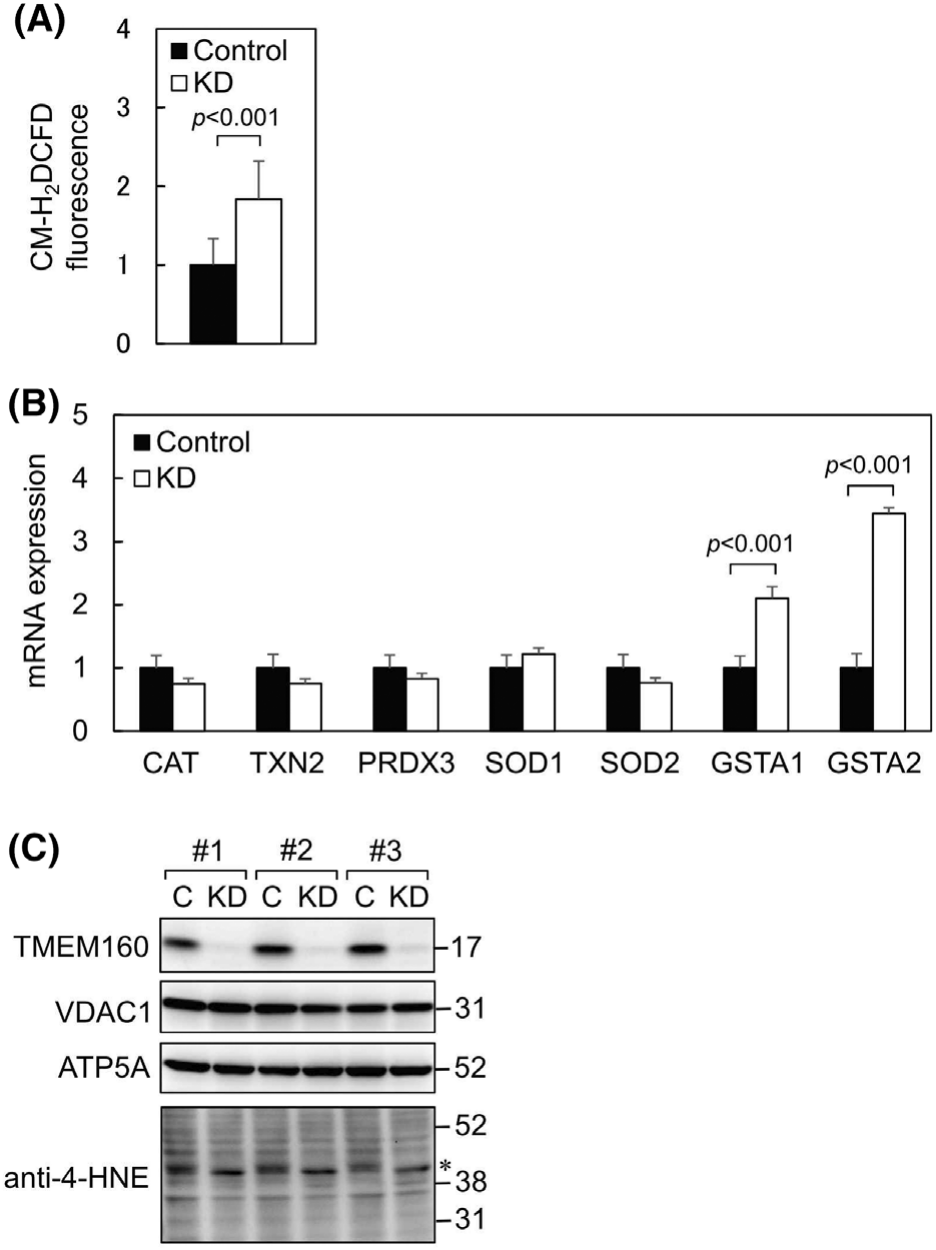
Depletion of TMEM160 led to increased ROS generation and upregulation of GST genes and likely enhanced protein degradation. HepG2 cells were transfected with control siRNA and TMEM160‐targeted siRNA. (A) Fluorescence intensity of CM‐H_2_DCFDA‐loaded cells was measured using an Infiniti F200 pro (*n* = 48). Data were analyzed using Student's *t*‐test, and the error bars represent mean ± standard deviation (SD). The levels of CM‐H_2_DCFDA fluorescence in the control were set at 1. (B) mRNA levels of ROS‐detoxifying enzymes and GSTs metabolizing the secondary products yielded by ROS were measured using quantitative PCR (*n* = 4). Data were analyzed using Student's *t*‐test, and the error bars represent mean ± SD. mRNA levels of the controls were considered as 1 [CAT, catalase; GSTA1 and GSTA2, glutathione *S*‐transferase alpha 1 and 2; PRDX3, peroxiredoxin 3; SOD1 and SOD2, superoxide dismutase 1 and 2; TXN2, thioredoxin 2]. (C) Proteins extracted from the cells were subjected to immunoblot analysis to determine the expression levels of the indicated proteins as well as to detect 4‐HNE‐modified proteins (*n* = 3).

Upregulation of HSPD1, TOMM22, and TOMM20 suggests that TMEM160 depletion causes mitochondrial damage. Damage to the mitochondrial electron transport chain (ETC) results in an enhanced release of ROS from the ETC. Therefore, we quantified ROS in cells using CM‐H_2_DCFDA (Fig. 5A). CM‐H_2_DCFDA fluorescence (green fluorescence) was significantly increased in TMEM160‐depleted cells, indicating that TMEM160 depletion led to increased ROS generation. To examine whether oxidative stress is induced by TMEM160 depletion, we measured the mRNA levels of ROS‐detoxifying enzymes (Fig. 5B). Although the mRNA levels of ROS‐detoxifying enzymes (CAT, TXN2, PRDX3, SOD1, and SOD2) were unchanged, the levels of GST alpha (GSTA1 and GSTA2), which metabolize the secondary products of oxidative stress, were increased after TMEM160 depletion. It is well‐known that increased ROS generation enhances the peroxidation of lipids and that the resulting peroxidated lipids release 4‐HNE, which modifies proteins and DNA [3]. Therefore, we performed immunoblot analysis using anti‐4‐HNE antibody to detect 4‐HNE‐modified proteins (Fig. 5C). The patterns on the immunoblot image of 4‐HNE‐modified proteins differed between the control and TMEM160‐depleted cell lysates. Particularly, the strongest band detected using an anti‐4‐NHE antibody in control cells was shifted to lower size, indicating that protein degradation was enhanced by TMEM160 depletion. Taken together, TMEM160 depletion led to increased ROS generation, increased levels of GSTs, and enhanced protein degradation.

### NAC treatment reduces intracellular ROS levels but does not reduce both UPR^mt^ and the enhanced expression of TOMM22

**Fig. 6:**
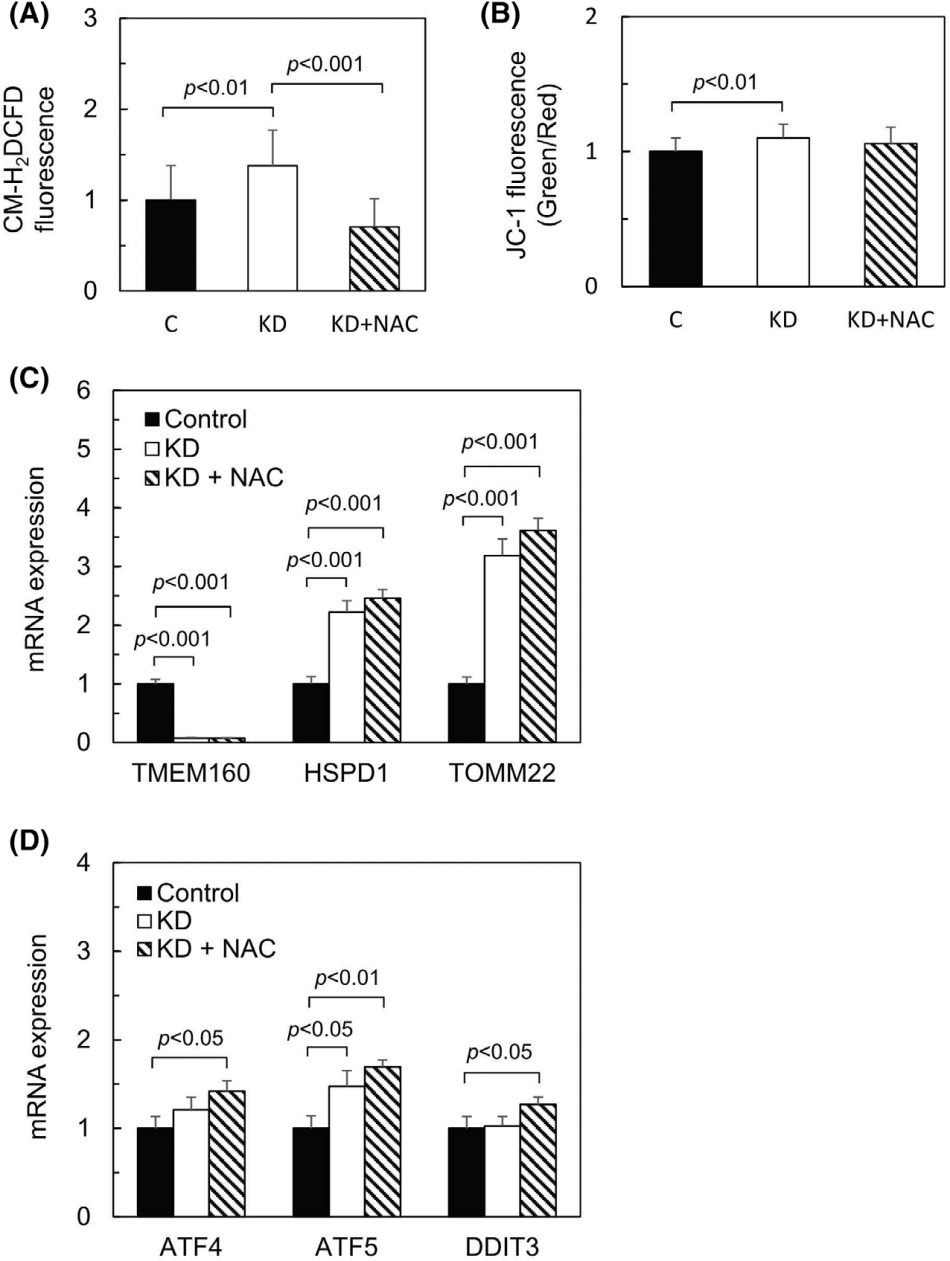
*N*‐acetylcysteine treatment reduces intracellular ROS levels but does not reduce both UPR^mt^ and the enhanced expression of TOMM22. HepG2 cells were transfected with control siRNA and TMEM160‐targeted siRNA. After 24 h of siRNA transfection, the cells were treated with or without 10 mm NAC (pH 7.5) and then further cultured for 48 h. all data were analyzed using Student's *t*‐test, and the error bars represent mean ± standard deviation (SD). (A) Fluorescence intensity of CM‐H_2_DCFDA‐loaded cells was measured using Infiniti F200 pro (*n* = 16). The levels of CM‐H_2_DCFDA fluorescence in the control were set at 1. (B) Intensity of both red and green fluorescence of JC‐1‐loaded cells was measured using Infiniti F200 pro, and the ratio of green fluorescence to red fluorescence (green/red) was calculated (*n* = 16). The ratio of the fluorescence in the control was set at 1. (C) mRNA levels of TMEM160, HSPD1, and TOMM22 were measured using quantitative PCR (*n* = 3). mRNA levels of the controls were considered as 1. (D) mRNA levels of ATF4, ATF5, and DDIT3 were measured using quantitative PCR (*n* = 3). mRNA levels of the controls were considered as 1.

We next examined whether the ROS scavenger NAC could reduce the UPR^mt^ and the enhanced expression of TOMM22 induced by TMEM160 depletion (Fig. 6). After 24 h of siRNA transfection, TMEM160‐depleted cells were treated with or without NAC for 48 h. We found that ROS, which had been upregulated by TMEM160 depletion, was reduced by NAC treatment (Fig. 6A). To examine whether TMEM160 depletion induces apoptosis, the cells were treated with JC‐1 and the fluorescence intensity in living cells (red fluorescence) and apoptotic cells (green fluorescence) was measured. A small but significant increase in the ratio of green to red fluorescence (green/red) was observed (Fig. 6B), suggesting that TMEM160 depletion slightly induces apoptosis. We further analyzed the mRNA levels of HSPD1 and TOMM22 in TMEM160‐depleted cells (Fig. 6C). Unexpectedly, the expression of HSPD1 and TOMM22, which had been enhanced by TMEM160 depletion, was not surpressed in NAC‐treated cells (Fig. 6C). The mRNA levels of ATF4, ATF5, and DDIT3 were also not reduced by subsequent NAC treatment in TMEM160‐depleted cells (Fig. 6D). These results indicate that induction of the UPR^mt^ and upregulation of TOMM22 by TMEM160 depletion is not suppressed by the subsequent detoxification of ROS.

## Discussion

In this study, we determined the mitochondrial localization of TMEM160 (see Figs 1 and 2). Mitochondria are divided by outer and inner membranes into four compartments – outer membrane, intermembrane space, inner membrane, and matrix [9]. Most mitochondrial proteins are encoded by nuclear DNA and are synthesized as precursors in the cytosol and then imported into mitochondria [9, 10, 11]. These proteins are recognized by the translocase of the outer membrane (TOM) complex and then translocated through the translocase of the inner membrane (TIM) complex. The TOM complex is composed of several proteins such as TOMM22, TOMM20, and TOMM40. TOMM22, a receptor of the TOM complex, has an additional function in organizing TOM complex formation using the pore‐forming protein TOMM40 [12]. Precursors translocated through the TOM and TIM complexes are pulled into the matrix by HSPA9 (mitochondrial Hsp70) [9].

TMEM160 localizes to the mitochondrial inner membrane, and TMEM160 contributes to the establishment of discrete nerve injury‐induced pain behaviors in male mice [1]. Although we performed an immunostaining assay for endogenous TMEM160 in HepG2, HeLa, and COS‐7 cells using an anti‐TMEM160 antibody, we failed to detect the protein. Therefore, we confirmed the mitochondrial localization of TMEM160 using cells stably expressing TMEM160 with a DYKDDDDK‐tag at the C‐terminus. As the C‐terminal portion of TMEM160 is topologically expected to face the intermembrane‐space, an antibody against DYKDDDDK would be accessible to the tag. Integration of TMEM160 into the mitochondrial membrane was confirmed using the alkali‐extraction method, as previously reported [4]. Furthermore, the mitochondrial inner‐membrane localization of TMEM160 was confirmed using the digitonin extraction assay; digitonin specifically permeabilizes mitochondrial outer membrane, leaving the cardiolipin‐rich inner membrane rich intact. These results indicate that TMEM160 resides in the mitochondrial inner membrane or matrix. Taken together, our results confirm previous findings showing that TMEM160 is a transmembrane protein of the mitochondrial inner membrane [1].

Mitochondria play important roles in ATP generation. The ETC comprising four complexes (complexes I, II, III, and IV) is coupled with ATP synthase to generate ATP, and oxygen is consumed in complex IV [13, 14]. However, when the ETC is disrupted, electrons leak from the ETC and react with oxygen to generate ROS [15]. ROS can damage DNA, membrane lipids, and proteins, and cells through oxidative stress. Under oxidative stress conditions, ROS‐detoxifying enzymes and enzymes that metabolize secondary products of ROS are induced to prevent cell damage [16, 17]. In addition, the UPR^mt^ is activated when damaged or misfolded proteins accumulate in the mitochondria [18, 19, 20]. During the UPR^mt^, nuclear‐encoded proteins (such as mitochondrial chaperones and proteases) are imported into the mitochondria. Chaperones facilitate the proper folding of proteins, and proteases remove damaged or misfolded proteins [21, 22]. Additionally, some proteins, such as TOMM20, TOMM22 (mitochondrial protein import receptors), and TIMM17 (translocase of the inner membrane subunit), which are involved in mitochondrial protein import, are induced during the UPR^mt^ [8, 23, 24].

To assess the importance of TMEM160 in mitochondrial maintenance, we knocked down TMEM160 in cultured cells. Depletion of TMEM160 led to upregulation of the UPR^mt^‐related protein HSPD1 and the UPR^mt^‐inducing transcription factors ATF4, ATF5, and DDIT3. In addition, upregulation of the mitochondrial protein import receptors TOMM22 and TOMM20 was observed following TMEM160 depletion. Numerous proteins have been reported to be induced by UPR^mt^ [2, 25]; these include mitochondrial import‐related proteins such as TOMM20 [23, 24]. Notably, TOMM22 is upregulated by TMEM160 depletion as well as by TMEM65 depletion [8]. In addition to serving as an import receptor, TOMM22 acts as an organizer of the TOM complex, which contains several proteins, including TOMM40 and TOMM20 [12]. Increased levels of TOMM22 may enhance formation of the TOM complex and facilitate protein import into mitochondria to partially alleviate the mitochondrial damage induced by TMEM160 depletion. Indeed, a recent study reported that mitochondrial protein import is enhanced at an early stage of the UPR^mt^ [26]. Thus, TMEM160 depletion may result in important but not strong upregulation of the UPR^mt^ and TOMM22 to prevent mitochondrial damage.

We found that TMEM160 depletion significantly increased ROS generation, indicating that TMEM160 surpresses ROS generation. Increased ROS generation can damage proteins. The asterisk‐marked bands mainly detected in TMEM160‐depeleted cell lysates, as shown in Fig. 3D, may correspond to the degradation products of HSPD1. In addition, the asterisk‐marked major band detected by anti‐4‐HNE antibody in TMEM160‐depeleted cell lysates, as shown in Fig. 5C, may correspond to a degraded 4‐HNE‐modified protein. This observation indicates that TMEM160 may stabilize some mitochondrial protein(s); however, this hypothesis requires verification. This prediction is supported by the observation that subsequent detoxification of ROS with NAC did not reduce the UPR^mt^ (Fig. 6C,D). Together, TMEM160 may stabilize and prevent the aggregation of some types of mitochondrial protein(s).

Analysis of the mRNA levels of redox‐related genes showed that two GSTs (GSTA1 and GSTA2) were upregulated in TMEM160‐depleted cells. As GSTs metabolize the products of oxidative stress [17], they may also protect the mitochondria from oxidative stress induced by TMEM160 depletion. However, it remains unclear why the levels of other antioxidative enzymes tested (CAT, TXN2, PRDX3, SOD1, and SOD2) remained unchanged. It is possible that induction of the UPR^mt^, upregulation of the mitochondrial protein import receptors TOMM22 and TOMM20, and upregulation of GSTs may be sufficient to rescue the mitochondria weakly damaged by TMEM160 depletion. Indeed, only a slight increase in the number of apoptotic cells was observed after TMEM160 depletion (Fig. 6B). Therefore, TMEM160‐depleted mitochondria may appear to be normal, as reported previously [1].

## Conclusions

Although further investigation is necessary to reveal the precise role of TMEM160, we showed that depletion of TMEM160 leads to ROS generation, enhances protein degradation, induces the UPR^mt^, upregulates the mitochondrial protein import receptors TOMM22 and TOMM20, and upregulates GSTs. Additionally, TMEM160 may not only surpress ROS generation but also stabilize and prevent the aggregation of some types of mitochondrial proteins.

## Conflict of interest

The authors declare no conflict of interest.

## Author contributions

KY performed experiments. MH analyzed data. MY performed experiments, analyzed data, and wrote this article.

## Supporting information


**Fig. S1.** A second TMEM160‐targeted siRNA#2 (siRNA#2) also significantly reduces mRNA expression of TMEM160 and upregulates mRNA expression of HSPD1 and TOMM22. HepG2 cells were transfected with control siRNA and the TMEM160‐targeted siRNA#2. (A) The RNA sequence of siRNA#2 is shown. The sequence of siRNA#2 is not overlapped with the TMEM160‐targeted siRNA described in “Materials and methods.” (B) mRNA level of TMEM160 were examined by quantitative PCR (*n* = 3). Data were analyzed using Student's *t*‐test, and the error bars represent mean ± standard deviation (SD). The mRNA levels of the control were set as 1. (C) mRNA levels of HSPD1 and TOMM22 were examined by quantitative PCR (*n* = 3). Data were analyzed using Student's *t*‐test, and the error bars represent mean ± SD. The mRNA levels of the control were set as 1.

**Fig. S2.** Morphological change in mitochondria was not observed by TMEM160 depletion. HepG2 cells cultured on cover grass were transfected with control siRNA (Control) and TMEM160‐targeted siRNA (KD). After 72 h incubation, these cells were stained with MitoTracker Red, and followed by Hoechst stain. Photographs of MitoTracker Red (red fluorescence) and Hoechst (blue fluorescence) under fluorescence microscope, and the merged ones, are shown. Scale bar, 10 μm.

**Table S1.** Primers used for quantitative PCR.

## Data Availability

The data that support the findings of this study are available from the corresponding author (yano@kumamoto-hsu.ac.jp) upon reasonable request.
