# Supplementary figures and images for: Knockdown of TMEM160 leads to an increase in reactive oxygen species generation and the induction of the mitochondrial unfolded protein response
Source: FEBS Open Bio. 2022 Oct 20;12(12):2179–90. doi: 10.1002/2211-5463.13496 (PMC9714381; doi:10.1002/2211-5463.13496)

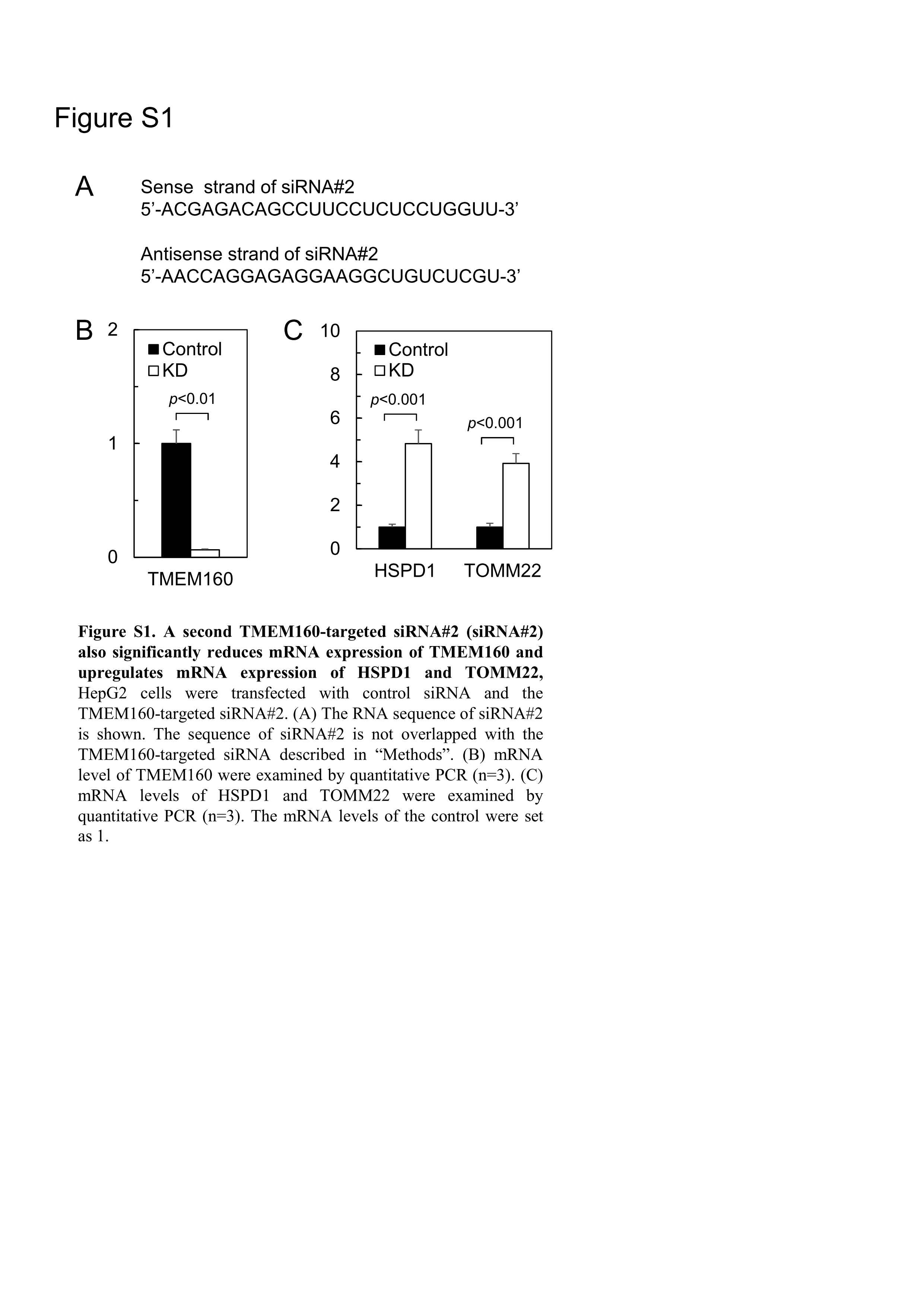

Supplement: Supplementary file 1 — Fig. S1. A second TMEM160‐targeted siRNA#2 (siRNA#2) also significantly reduces mRNA expression of TMEM160 and upregulates mRNA expression of HSPD1 and TOMM22. HepG2 cells were transfected with control siRNA and the TMEM160‐targeted siRNA#2. (A) The RNA sequence of siRNA#2 is shown. The sequence of siRNA#2 is not overlapped with the TMEM160‐targeted siRNA described in “Materials and methods.” (B) mRNA level of TMEM160 were examined by quantitative PCR (n = 3). Data were analyzed using Student's t‐test, and the error bars represent mean ± standard deviation (SD). The mRNA levels of the control were set as 1. (C) mRNA levels of HSPD1 and TOMM22 were examined by quantitative PCR (n = 3). Data were analyzed using Student's t‐test, and the error bars represent mean ± SD. The mRNA levels of the control were set as 1. [file FEB4-12-2179-s002.jpg]

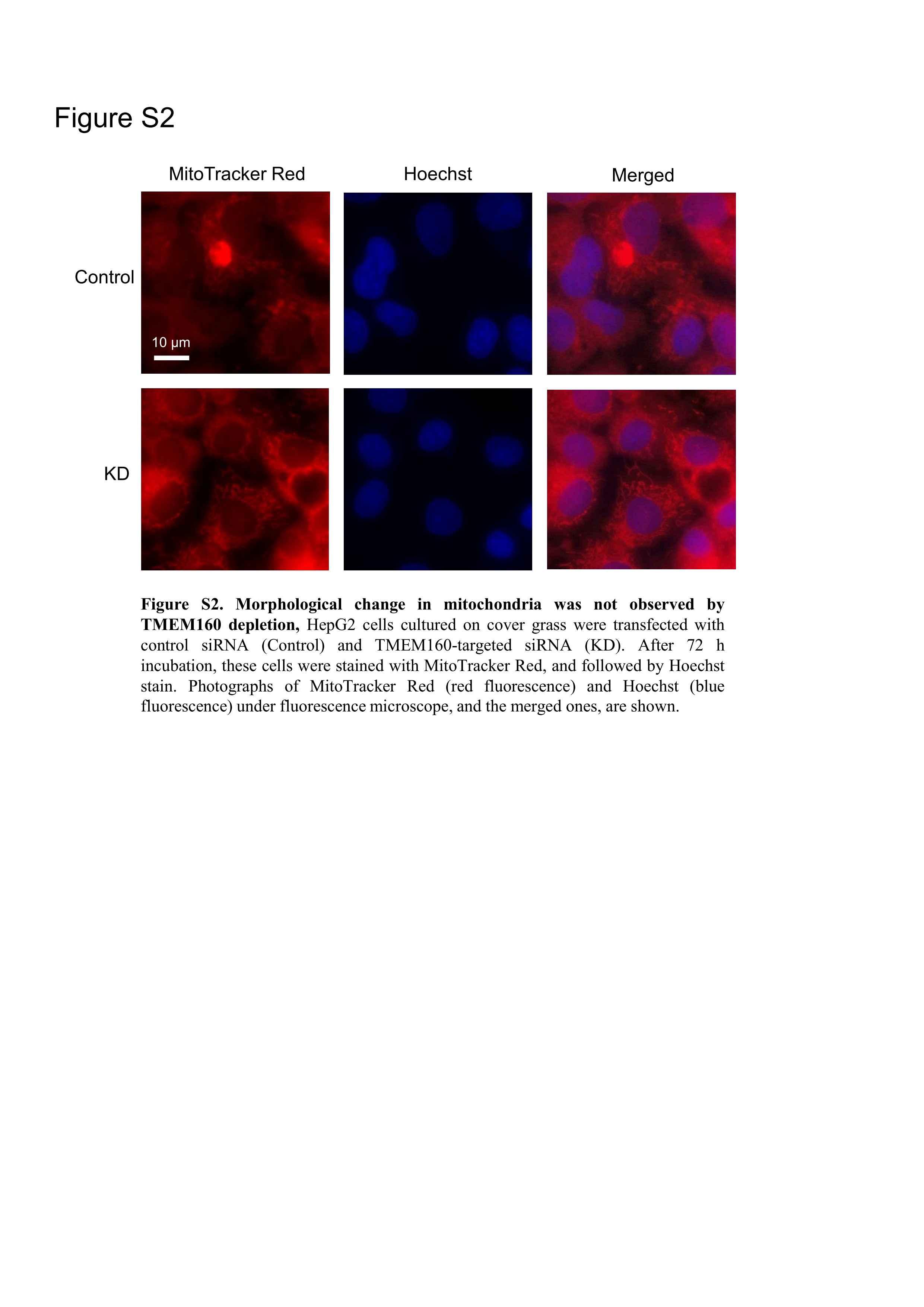

Supplement: Supplementary file 2 — Fig. S2. Morphological change in mitochondria was not observed by TMEM160 depletion. HepG2 cells cultured on cover grass were transfected with control siRNA (Control) and TMEM160‐targeted siRNA (KD). After 72 h incubation, these cells were stained with MitoTracker Red, and followed by Hoechst stain. Photographs of MitoTracker Red (red fluorescence) and Hoechst (blue fluorescence) under fluorescence microscope, and the merged ones, are shown. Scale bar, 10 μm. [file FEB4-12-2179-s003.jpg]
