# Supplementary material for: Knockdown of TMEM160 leads to an increase in reactive oxygen species generation and the induction of the mitochondrial unfolded protein response
Source: FEBS Open Bio. 2022 Oct 20;12(12):2179–90. doi: 10.1002/2211-5463.13496 (PMC9714381; doi:10.1002/2211-5463.13496)
Supplement: Supplementary file 3 — Table S1. Primers used for quantitative PCR. [file FEB4-12-2179-s001.docx]

| Supplementary Table 1. Primers used for quantitative PCR | | |
| --- | --- | --- |
| Target gene | Forward primer | Reverse primer |
| GAPDH | 5'-TGACAACAGCCTCAAGAT-3' | 5'-GAGTCCTTCCACGATACC-3' |
| HSPD1 | 5'-CAAAGGTGCTAATCCAGTGGAAATC-3' | 5'-TCTTTGTCTCCGTTTGCAGAAATC-3' |
| LONP1 | 5'-GGGACGTTTGCCCAGATCC-3' | 5'-TCTTGCCCCGCTTTGACTTC-3' |
| TOMM22 | 5'-CAGTCCCCGGACGAATTGC-3' | 5'-CGACAGGGTCTCATCTAGCTC-3' |
| CAT | 5'-TCCAAGGCAAAGGTATTTGAGCA-3' | 5'-CAACGAGATCCCAGTTACCATCTTC-3' |
| TXN2 | 5'-AATATCCAGGATGGACCTGACTTTC-3' | 5'-AGGATCTTGCAGGGTCCACA-3' |
| PRDX3 | 5'-GAAGTTGTCGCAGTCTCAGTGG-3' | 5'-CAAGAGTGCGATGTTCATGTGG-3' |
| SOD1 | 5'-GGTGGGCCAAAGGATGAAGAG-3' | 5'-CCACAAGCCAAACGACTTCC-3' |
| SOD2 | 5'-CCAAATCAGGATCCACTGCAA-3' | 5'-CAGCATAACGATCGTGGTTTACTT-3' |
| GSTA1 | 5'-AGCCGGGCTGACATTCATCT-3' | 5'-TGGCCTCCATGACTGCGTTA-3' |
| GSTA2 | 5'-GCAGACCAGAGCCATTCTCAACTAC-3' | 5'-GGCAAGCTTGGCATCTTGTTC-3' |
| TMEM160 | 5'-TCCGAAAAGCGCACGAGA-3' | 5'-TCTGCATGAAGGAGATGACCC-3' |
| ATF4 | 5'-ATGACCGAAATGAGCTTCCTG-3' | 5'-GCTGGAGAACCCATGAGGT-3' |
| ATF5 | 5'-AGGGGACCGCAAGCAAAAG-3' | 5'-GCCTTGTAAACCTCGATGAGC-3' |
| DDIT3 | 5'-GGAAACAGAGTGGTCATTCCC-3' | 5'-CTGCTTGAGCCGTTCATTCTC-3' |
